# Supplementary material for: Regulation of life span by the gut microbiota in the short-lived African turquoise killifish
Source: eLife. 2017 Aug 22;6:e27014. doi: 10.7554/eLife.27014 (PMC5566455; doi:10.7554/eLife.27014)
Supplement: Figure 1—source data 1. — For each species indicated in the first row, the values in column display the rank order of each bacterial phylum indicated in bold in the first column. DOI: http://dx.doi.org/10.7554/eLife.27014.004 [file elife-27014-fig1-data1.docx]

**Figure 1 – source data 1**

| **Ranked abundance of bacterial phyla shared with the turquoise killifish** | | | | |
| --- | --- | --- | --- | --- |
|  |  |  |  |  |
|  | **TK** | **Zebrafish** | **Mouse** | **Human** |
| **Proteobacteria** | 1 | 1 | 4 | 4 |
| **Firmicutes** | 2 | 5 | 2 | 1 |
| **Actinobacteria** | 3 | 6 | 5 | 3 |
| **Bacteroidetes** | 4 | 4 | 1 | 2 |
| **Fusobacteria** | 5 | 2 | NA | 10 |
| **Cyanobacteria** | 6 | 9 | 7 | 8 |
| **Planctomycetes** | 7 | 7 | NA | NA |
| **Chloroflexi** | 8 | 12 | NA | NA |
| **Verrucomicrobia** | 9 | 8 | 6 | 7 |
| **TM7** | 10 | 11 | 8 | 9 |
| **Acidobacteria** | 11 | 13 | NA | 11 |
| **SBR1093** | 12 | NA | NA | NA |
| **Euryarchaeota** | 13 | NA | NA | 6 |
| **Armatimonadetes** | 14 | NA | NA | 12 |
| **Spirochaetes** | 15 | NA | NA | NA |
| **TM6** | 15 | NA | NA | NA |
| **Nitrospirae** | 16 | 10 | NA | NA |
| **Tenericutes** | 16 | 3 | 3 | 5 |
| **[Thermi]** | 17 | 15 | 9 | NA |
| **Chlamydiae** | 18 | 14 | NA | NA |
| **WPS-2** | 19 | NA | NA | NA |
