# Supplementary material for: Regulation of life span by the gut microbiota in the short-lived African turquoise killifish
Source: eLife. 2017 Aug 22;6:e27014. doi: 10.7554/eLife.27014 (PMC5566455; doi:10.7554/eLife.27014)
Supplement: Figure 1—source data 2. — A indicates Archaea, B indicates Bacteria. DOI: http://dx.doi.org/10.7554/eLife.27014.005 [file elife-27014-fig1-data2.docx]

| **Figure 1 – source data 2**  **Relative phylum abundance. A: Archaea; B: Bacteria** | | |  |  |
| --- | --- | --- | --- | --- |
|  |  |  |  |  |
|  | **Human** | **TK** | **Mouse** | **Zebrafish** |
| **Crenarchaeota (A)** | 0.0 | 0.0 | 0.0 | 1.33E-06 |
| **Euryarchaeota (A)** | 2.35E-03 | 6.06E-06 | 0.0 | 0.0 |
| **Acidobacteria (B)** | 2.33E-07 | 1.63E-05 | 0.0 | 1.86E-05 |
| **Actinobacteria (B)** | 2.59E-02 | 9.43E-02 | 1.59E-03 | 6.05E-03 |
| **Armatimonadetes (B)** | 1.17E-07 | 5.13E-06 | 0.0 | 0.0 |
| **Bacteroidetes (B)** | 2.13E-01 | 1.15E-02 | 5.00E-01 | 7.00E-02 |
| **Chlamydiae (B)** | 0.0 | 9.33E-07 | 0.0 | 9.30E-06 |
| **Chlorobi (B)** | 2.33E-07 | 0.0 | 0.0 | 1.33E-06 |
| **Chloroflexi (B)** | 0.0 | 2.42E-04 | 0.0 | 2.13E-05 |
| **Cyanobacteria (B)** | 3.82E-05 | 4.47E-03 | 2.74E-05 | 8.24E-05 |
| **Deferribacteres (B)** | 0.0 | 0.0 | 5.34E-03 | 0.0 |
| **Firmicutes (B)** | 7.40E-01 | 2.02E-01 | 4.71E-01 | 7.09E-03 |
| **Fusobacteria (B)** | 5.02E-06 | 8.60E-03 | 0.0 | 2.05E-01 |
| **Gemmatimonadetes (B)** | 1.17E-07 | 0.0 | 0.0 | 0.0 |
| **Lentisphaerae (B)** | 2.31E-05 | 0.0 | 0.0 | 0.0 |
| **Nitrospirae (B)** | 0.0 | 1.87E-06 | 0.0 | 6.51E-05 |
| **OD1 (B)** | 0.0 | 0.0 | 0.0 | 1.33E-06 |
| **Planctomycetes (B)** | 0.0 | 1.04E-03 | 0.0 | 2.34E-04 |
| **Proteobacteria (B)** | 9.07E-03 | 6.78E-01 | 4.75E-03 | 5.49E-01 |
| **SBR1093 (B)** | 0.0 | 9.33E-06 | 0.0 | 0.0 |
| **SR1 (B)** | 0.0 | 0.0 | 0.0 | 0.0 |
| **Spirochaetes (B)** | 0.0 | 4.20E-06 | 0.0 | 0.0 |
| **Synergistetes (B)** | 4.03E-05 | 0.0 | 0.0 | 0.0 |
| **TM6 (B)** | 0.0 | 4.20E-06 | 0.0 | 0.0 |
| **TM7 (B)** | 1.03E-05 | 3.36E-05 | 1.30E-05 | 2.92E-05 |
| **Tenericutes (B)** | 6.99E-03 | 1.87E-06 | 1.75E-02 | 1.62E-01 |
| **Verrucomicrobia (B)** | 2.06E-03 | 8.49E-05 | 2.88E-05 | 1.35E-04 |
| **WPS-2 (B)** | 0.0 | 4.66E-07 | 0.0 | 0.0 |
| **[Thermi] (B)** | 0.0 | 1.40E-06 | 7.20E-06 | 3.98E-06 |
