# Supplementary material for: Regulation of life span by the gut microbiota in the short-lived African turquoise killifish
Source: eLife. 2017 Aug 22;6:e27014. doi: 10.7554/eLife.27014 (PMC5566455; doi:10.7554/eLife.27014)
Supplement: Figure 2—source data 1. — DOI: http://dx.doi.org/10.7554/eLife.27014.007 [file elife-27014-fig2-data1.docx]

| **Figure 2 – source data 1**  **Collection points of the wild fish populations** | | |
| --- | --- | --- |
|  |  |  |
| **Abbreviation** | **Strain name** | **GPS coordinates** |
| - | Z-GNP#00 | S21˚ 48.8724’ E31˚ 55.9332’ |
| Z | Z-GNP#01 | S21˚ 48.198’ E31˚ 55.2306’ |
| - | Z-GNP#02 | S21˚ 48.1836’ E31˚ 55.2342’ |
| - | Z-GNP#03 | S21˚ 48.1512’ E31˚ 55.2756’ |
| - | Z-GNP#04 | S21˚ 46.4358’ E31˚ 52.9494’ |
| M1 | M-PNB#01 | S22˚ 33.2778’ E32˚ 43.635’ |
| M2 | M-LNP#01 | S24˚ 2.2848’ E32˚ 26.3592’ |
| - | M-LNP#02 | S24˚ 8.4156’ E32˚ 15.0816’ |
| - | M-LNP#03 | S24˚ 15.0756’ E32˚ 28.0428’ |
