# Supplementary material for: Regulation of life span by the gut microbiota in the short-lived African turquoise killifish
Source: eLife. 2017 Aug 22;6:e27014. doi: 10.7554/eLife.27014 (PMC5566455; doi:10.7554/eLife.27014)
Supplement: Figure 2—source data 2. — DOI: http://dx.doi.org/10.7554/eLife.27014.008 [file elife-27014-fig2-data2.docx]

| **Figure 2 – source data 2**  **Ecological factors associated with wild fish populations** | | | | |  |  |  |
| --- | --- | --- | --- | --- | --- | --- | --- |
|  |  |  |  |  |  |  |  |
| **Location** | **Altitude  (m)** | **Size  (m2)** | **Conductivity  (microS per cm2)** | **Water temp.  (C)** | **Turbidity  (1 highest, 4 lowest)** | **Maximum pool depth  (cm)** | **Littoral vegetation  (coverage in %)** |
| M_PNB#01 | 105.5 | 400 | 80 | 23 | 4 | 30 | 0 |
| M_LNP#01 | 79.5 | 250 | 330 | 33.7 | 2 | 20 | 100 |
| M_LNP#02 | 119.5 | 280 | 140 | 34 | 3 | 100 | 5 |
| M_LNP#03 | 49.6 | 3500 | 35 | 29 | 2 | 50 | 100 |
| Z_GNP#00 | 320.3 | NA | NA | NA | NA | NA | NA |
| Z_GNP#01 | 324.6 | NA | NA | NA | NA | NA | NA |
| Z_GNP#02 | 325.4 | NA | NA | NA | NA | NA | NA |
| Z_GNP#03 | 326.3 | NA | NA | NA | NA | NA | NA |
| Z_GNP#04 | 339.6 | NA | NA | NA | NA | NA | NA |
