# Supplementary material for: Regulation of life span by the gut microbiota in the short-lived African turquoise killifish
Source: eLife. 2017 Aug 22;6:e27014. doi: 10.7554/eLife.27014 (PMC5566455; doi:10.7554/eLife.27014)
Supplement: Figure 4—source data 1. — DOI: http://dx.doi.org/10.7554/eLife.27014.014 [file elife-27014-fig4-data1.docx]

**Figure 4 – source file 1**

| **Transfer to 9.5-week-old fish** | | | | | |
| --- | --- | --- | --- | --- | --- |
| **Fish ID** | **Days** | **Omt** | **Ymt** | **wt** | **Abx** |
| 399 | 113 |  |  | 1 |  |
| 400 | 115 |  |  | 1 |  |
| 401 | 79 |  |  | 1 |  |
| 402 | 115 |  |  | 1 |  |
| 403 | 101 |  |  | 1 |  |
| 404 | 85 |  |  | 1 |  |
| 405 | 115 |  |  | 1 |  |
| 550 | 52 |  |  | 1 |  |
| 553 | 132 |  |  | 1 |  |
| 558 | 75 |  |  | 1 |  |
| 568 | 87 |  |  | 1 |  |
| 571 | 125 |  |  | 1 |  |
| 576 | 69 |  |  | 1 |  |
| 579 | 125 |  |  | 1 |  |
| 602 | 107 |  |  | 1 |  |
| 562 | 129 |  |  | 1 |  |
| 567 | 125 |  |  | 1 |  |
| 703 | 81 |  |  | 1 |  |
| 705 | 120 |  |  | 1 |  |
| 707 | 141 |  |  | 1 |  |
| 708 | 132 |  |  | 1 |  |
| 709 | 145 |  |  | 1 |  |
| 747 | 142 |  |  | 1 |  |
| 2 | 70 |  |  | 1 |  |
| 699 | 72 |  |  | 1 |  |
| 696 | 90 |  |  | 1 |  |
| 703 | 79 |  |  | 1 |  |
| 746 | 102 |  |  | 1 |  |
| 927 | 119 |  |  | 1 |  |
| 909 | 67 |  |  | 1 |  |
| 918 | 126 |  |  | 1 |  |
| 929 | 112 |  |  | 1 |  |
| 910 | 145 |  |  | 1 |  |
| 911 | 147 |  |  | 1 |  |
| 912 | 116 |  |  | 1 |  |
| 931 | 160 |  |  | 1 |  |
| 914 | 89 |  |  | 1 |  |
| 932 | 150 |  |  | 1 |  |
| 916 | 90 |  |  | 1 |  |
| 924 | 130 |  |  | 1 |  |
| 933 | 131 |  |  | 1 |  |
| 925 | 155 |  |  | 1 |  |
| 922 | 130 |  |  | 1 |  |
| 923 | 137 |  |  | 1 |  |
| 1 | 76 |  |  | 1 |  |
| 917 | 80 |  |  | 1 |  |
| 943 | 78 |  |  | 1 |  |
| 906 | 77 |  |  | 1 |  |
| 930 | 107 |  |  | 1 |  |
| 926 | 76 |  |  | 1 |  |
| 944 | 109 |  |  | 1 |  |
| 904 | 98 |  |  | 1 |  |
| 905 | 70 |  |  | 1 |  |
| 939 | 98 |  |  | 1 |  |
| 308 | 145 |  |  |  | 1 |
| 333 | 105 |  |  |  | 1 |
| 317 | 150 |  |  |  | 1 |
| 386 | 135 |  |  |  | 1 |
| 400 | 125 |  |  |  | 1 |
| 415 | 171 |  |  |  | 1 |
| 486 | 109 |  |  |  | 1 |
| 500 | 90 |  |  |  | 1 |
| 407 | 130 |  |  |  | 1 |
| 695 | 125 |  |  |  | 1 |
| 697 | 148 |  |  |  | 1 |
| 701 | 123 |  |  |  | 1 |
| 702 | 132 |  |  |  | 1 |
| 704 | 143 |  |  |  | 1 |
| 706 | 160 |  |  |  | 1 |
| 750 | 132 |  |  |  | 1 |
| 698 | 81 |  |  |  | 1 |
| 749 | 79 |  |  |  | 1 |
| 748 | 115 |  |  |  | 1 |
| 1278 | 72 |  |  |  | 1 |
| 1299 | 90 |  |  |  | 1 |
| 1100 | 72 |  |  |  | 1 |
| 379 | 93 |  | 1 |  |  |
| 380 | 147 |  | 1 |  |  |
| 381 | 115 |  | 1 |  |  |
| 382 | 80 |  | 1 |  |  |
| 383 | 157 |  | 1 |  |  |
| 386 | 183 |  | 1 |  |  |
| 387 | 168 |  | 1 |  |  |
| 388 | 122 | 1 |  |  |  |
| 389 | 168 | 1 |  |  |  |
| 391 | 91 | 1 |  |  |  |
| 392 | 75 | 1 |  |  |  |
| 393 | 71 | 1 |  |  |  |
| 394 | 130 | 1 |  |  |  |
| 396 | 180 |  | 1 |  |  |
| 397 | 165 |  | 1 |  |  |
| 395 | 141 |  | 1 |  |  |
| 504 | 82 | 1 |  |  |  |
| 505 | 118 | 1 |  |  |  |
| 506 | 161 | 1 |  |  |  |
| 507 | 107 | 1 |  |  |  |
| 508 | 160 | 1 |  |  |  |
| 511 | 77 | 1 |  |  |  |
| 512 | 165 | 1 |  |  |  |
| 513 | 84 | 1 |  |  |  |
| 516 | 172 |  | 1 |  |  |
| 519 | 93 | 1 |  |  |  |
| 520 | 123 |  | 1 |  |  |
| 521 | 135 | 1 |  |  |  |
| 522 | 169 |  | 1 |  |  |
| 523 | 143 |  | 1 |  |  |
| 524 | 166 |  | 1 |  |  |
| 525 | 152 | 1 |  |  |  |
| 518 | 105 |  | 1 |  |  |
| 517 | 83 | 1 |  |  |  |
| 527 | 126 | 1 |  |  |  |
| 641 | 140 | 1 |  |  |  |
| 640 | 157 | 1 |  |  |  |
| 625 | 146 |  | 1 |  |  |
| 626 | 153 | 1 |  |  |  |
| 621 | 169 |  | 1 |  |  |
| 624 | 160 |  | 1 |  |  |
| 629 | 159 | 1 |  |  |  |
| 634 | 147 | 1 |  |  |  |
| 642 | 169 |  | 1 |  |  |
| 643 | 167 |  | 1 |  |  |
| 646 | 140 |  | 1 |  |  |
| 623 | 134 | 1 |  |  |  |
| 670 | 91 | 1 |  |  |  |
| 636 | 85 |  | 1 |  |  |
| 622 | 89 | 1 |  |  |  |
| 633 | 114 | 1 |  |  |  |
| 630 | 109 | 1 |  |  |  |
| 637 | 100 | 1 |  |  |  |
| 639 | 101 |  | 1 |  |  |
| 638 | 115 |  | 1 |  |  |
| 631 | 132 | 1 |  |  |  |
| 644 | 76 | 1 |  |  |  |
| 628 | 65 | 1 |  |  |  |
| 648 | 149 |  | 1 |  |  |
| 620 | 174 |  | 1 |  |  |
| 626b | 158 |  | 1 |  |  |
| 348 | 180 |  | 1 |  |  |
| 361 | 174 |  | 1 |  |  |
| 320 | 174 |  | 1 |  |  |
| 399 | 155 |  | 1 |  |  |
| 401 | 145 |  | 1 |  |  |
| 410 | 124 |  | 1 |  |  |
| 417 | 134 |  | 1 |  |  |
| 411 | 195 |  | 1 |  |  |
| 466 | 154 |  | 1 |  |  |
| 501 | 92 |  | 1 |  |  |
| 1276 | 89 | 1 |  |  |  |
| 1277 | 91 | 1 |  |  |  |
| 1018 | 81 | 1 |  |  |  |
| 1058 | 82 | 1 |  |  |  |
| 1017 | 96 |  | 1 |  |  |
| 1011 | 90 | 1 |  |  |  |
| 1040 | 83 | 1 |  |  |  |
| 1010 | 86 |  | 1 |  |  |
| 1016 | 108 | 1 |  |  |  |
| 1401 | 147 |  |  |  | 1 |
| 1405 | 138 |  |  |  | 1 |
| 1404 | 138 |  |  |  | 1 |
| 1421 | 148 |  |  |  | 1 |
| 1408 | 165 |  |  |  | 1 |
| 1428 | 168 |  |  |  | 1 |
| 1417 | 86 |  |  |  | 1 |
| 1420 | 86 |  |  |  | 1 |
| 1411 | 63 |  |  |  | 1 |
| 1424 | 86 |  |  |  | 1 |
| 1432 | 81 |  |  |  | 1 |
| 1410 | 113 |  |  |  | 1 |
| 1431 | 101 |  |  |  | 1 |
| 1427 | 92 |  |  |  | 1 |
| 1415 | 122 |  |  |  | 1 |
| 1423 | 108 |  |  |  | 1 |
| 14.02 | 150 |  |  |  | 1 |
| 1433 | 152 |  |  |  | 1 |
| 1426 | 145 |  |  |  | 1 |
| 1668 | 83 |  | 1 |  |  |
| 1674 | 151 |  | 1 |  |  |
| 1677 | 127 |  | 1 |  |  |
| 1696 | 118 |  | 1 |  |  |
| 1725 | 132 |  | 1 |  |  |
| 1750 | 135 |  | 1 |  |  |
| 1573 | 130 |  | 1 |  |  |
| 1570 | 114 |  | 1 |  |  |
| 1572 | 107 |  | 1 |  |  |
| 1545 | 171 |  | 1 |  |  |
| 1558 | 99 |  | 1 |  |  |
| 1562 | 85 |  | 1 |  |  |
| 1563 | 124 |  | 1 |  |  |
| 1675 | 131 |  | 1 |  |  |
| 1672 | 113 |  | 1 |  |  |
| 1673 | 85 | 1 |  |  |  |
| 1646 | 66 | 1 |  |  |  |
| 1645 | 70 | 1 |  |  |  |
| 1659 | 66 | 1 |  |  |  |
| 1662 | 116 | 1 |  |  |  |
| 1647 | 78 | 1 |  |  |  |
| 1688 | 155 | 1 |  |  |  |
| 1690 | 133 | 1 |  |  |  |
| 1672 | 106 | 1 |  |  |  |
| 1582 | 108 | 1 |  |  |  |
| 1702 | 90 | 1 |  |  |  |
| 1726 | 94 | 1 |  |  |  |
| 1727 | 99 | 1 |  |  |  |
| 1730 | 69 | 1 |  |  |  |
| 1580 | 115 | 1 |  |  |  |
| 1721 | 88 | 1 |  |  |  |
| 1722 | 115 | 1 |  |  |  |
| 1731 | 106 | 1 |  |  |  |
| 1772 | 105 |  |  |  | 1 |
| 1729 | 99 |  |  |  | 1 |
| 1679 | 67 |  |  |  | 1 |
| 1703 | 152 |  |  |  | 1 |
| 1694 | 95 |  |  |  | 1 |
| 1680 | 86 |  |  |  | 1 |
| 1686 | 140 |  |  |  | 1 |
| 1708 | 114 |  |  |  | 1 |
| 1665 | 121 |  |  |  | 1 |
| 1547 | 116 |  |  |  | 1 |
| 1551 | 106 |  |  |  | 1 |
| 1728 | 72 |  |  |  | 1 |
| 1574 | 118 |  |  |  | 1 |
| 1580 | 114 |  |  |  | 1 |
| 1786 | 69 |  |  | 1 |  |
| 1724 | 84 |  |  | 1 |  |
| 1803 | 89 |  |  | 1 |  |
| 1643 | 154 |  |  | 1 |  |
| 1785 | 107 |  |  | 1 |  |
| 1798 | 88 |  |  | 1 |  |
| 1666 | 115 |  |  | 1 |  |
| 1664 | 140 |  |  | 1 |  |
| 1667 | 122 |  |  | 1 |  |
| 1773 | 88 |  |  | 1 |  |
| 1792 | 115 |  |  | 1 |  |
| 1600 | 73 |  |  | 1 |  |
| 1687 | 95 |  |  | 1 |  |
| 1609 | 82 |  |  | 1 |  |
| 1702 | 136 |  |  | 1 |  |
| 1687 | 95 |  |  | 1 |  |
| 1648 | 128 |  |  | 1 |  |
