# Supplementary material for: Regulation of life span by the gut microbiota in the short-lived African turquoise killifish
Source: eLife. 2017 Aug 22;6:e27014. doi: 10.7554/eLife.27014 (PMC5566455; doi:10.7554/eLife.27014)
Supplement: Figure 4—source data 2. — DOI: http://dx.doi.org/10.7554/eLife.27014.015 [file elife-27014-fig4-data2.docx]

**Figure 4 – source data 2**

| **Transfer to 6-week old fish** | | | | | |
| --- | --- | --- | --- | --- | --- |
| **Fish ID** | **Days** | **Abx** | **wt** | **Old** | **6wk** |
| 1723 | 99 |  |  |  | 1 |
| 1640 | 101 |  |  |  | 1 |
| 1643 | 58 |  |  |  | 1 |
| 1716 | 72 |  |  |  | 1 |
| 1642 | 60 |  |  |  | 1 |
| 1769 | 82 |  |  |  | 1 |
| 1710 | 85 |  |  |  | 1 |
| 1720 | 84 |  |  |  | 1 |
| 1637 | 143 |  |  |  | 1 |
| 1717 | 83 |  |  |  | 1 |
| 1712 | 77 |  |  |  | 1 |
| 1715 | 79 |  |  |  | 1 |
| 1718 | 72 |  |  |  | 1 |
| 1638 | 130 |  |  |  | 1 |
| 1634 | 133 |  |  |  | 1 |
| 1635 | 133 |  |  |  | 1 |
| 1714 | 85 |  |  |  | 1 |
| 1773 | 93 |  |  |  | 1 |
| 1644 | 105 |  |  |  | 1 |
| 1641 | 103 |  |  |  | 1 |
| 1636 | 128 |  |  |  | 1 |
| 1639 | 109 |  |  |  | 1 |
| 1652 | 71 | 1 |  |  |  |
| 1704 | 58 | 1 |  |  |  |
| 1655 | 60 | 1 |  |  |  |
| 1706 | 46 | 1 |  |  |  |
| 1701 | 82 | 1 |  |  |  |
| 1656 | 105 | 1 |  |  |  |
| 1653 | 98 | 1 |  |  |  |
| 1699 | 82 | 1 |  |  |  |
| 1713 | 98 | 1 |  |  |  |
| 1703 | 72 | 1 |  |  |  |
| 1707 | 80 | 1 |  |  |  |
| 1771 | 101 | 1 |  |  |  |
| 1657 | 157 | 1 |  |  |  |
| 1658 | 154 | 1 |  |  |  |
| 1654 | 148 | 1 |  |  |  |
| 1649 | 149 | 1 |  |  |  |
| 1705 | 116 | 1 |  |  |  |
| 1650 | 137 | 1 |  |  |  |
| 1709 | 108 | 1 |  |  |  |
| 1651 | 106 | 1 |  |  |  |
| 1802 | 105 |  | 1 |  |  |
| 1806 | 100 |  | 1 |  |  |
| 1776 | 59 |  | 1 |  |  |
| 1797 | 75 |  | 1 |  |  |
| 1793 | 79 |  | 1 |  |  |
| 1778 | 78 |  | 1 |  |  |
| 1807 | 97 |  | 1 |  |  |
| 1800 | 95 |  | 1 |  |  |
| 1779 | 93 |  | 1 |  |  |
| 1784 | 130 |  | 0 |  |  |
| 1777 | 130 |  | 0 |  |  |
| 1775 | 130 |  | 0 |  |  |
| 1774 | 130 |  | 0 |  |  |
| 1734 | 118 |  | 1 |  |  |
| 1736 | 91 |  | 1 |  |  |
| 1757 | 75 |  | 1 |  |  |
| 1492 | 160 |  |  | 1 |  |
| 1533 | 133 |  |  | 1 |  |
| 1508 | 121 |  |  | 1 |  |
| 1495 | 120 |  |  | 1 |  |
| 1494 | 121 |  |  | 1 |  |
| 1487 | 116 |  |  | 1 |  |
| 1482 | 105 |  |  | 1 |  |
| 1490 | 102 |  |  | 1 |  |
| 1496 | 93 |  |  | 1 |  |
| 1547 | 95 |  |  | 1 |  |
| 1481 | 91 |  |  | 1 |  |
| 1480 | 92 |  |  | 1 |  |
| 1486 | 93 |  |  | 1 |  |
| 1479 | 76 |  |  | 1 |  |
| 1513 | 85 |  |  | 1 |  |
| 1491 | 74 |  |  | 1 |  |
| 1523 | 65 |  |  | 1 |  |
| 1485 | 49 |  |  | 1 |  |
| 1483 | 81 |  |  | 1 |  |
| 1489 | 55 |  |  | 1 |  |
| 1507 | 65 |  |  | 1 |  |
| 1503 | 135 |  |  | 1 |  |
| 1484 | 135 |  |  | 1 |  |
| 1493 | 135 |  |  | 1 |  |
| 1569 | 76 |  |  | 1 |  |
