# Supplementary material for: Regulation of life span by the gut microbiota in the short-lived African turquoise killifish
Source: eLife. 2017 Aug 22;6:e27014. doi: 10.7554/eLife.27014 (PMC5566455; doi:10.7554/eLife.27014)
Supplement: Figure 4—source data 3. — DOI: http://dx.doi.org/10.7554/eLife.27014.016 [file elife-27014-fig4-data3.docx]

| **Figure 4 – source data 3**  **Spontaneous Locomotor Activity (cm/20 min)** | | | | | |  |  |  |  |
| --- | --- | --- | --- | --- | --- | --- | --- | --- | --- |
| **6wk** | | | | **abx** | | **Omt** | | **ymt** | |
| **6wk** | **-1wk** | **+1wk** | **16wk** | **+1wk** | **16wk** | **+1wk** | **16wk** | **+1wk** | **16wk** |
| 4958 | 6709 | 3906 | 4220 | 3233 | 1501 | 7562 | 1681 | 9453 | 7321 |
| 6543 | 6130 | 3888 | 2674 | 1820 | 1500 | 5790 | 2886 | 7927 | 5219 |
| 1616 | 5089 | 3103 | 1397 | 5363 | 1889 | 4732 | 3182 | 3420 | 2638 |
| 7333 | 8300 | 4173 | 1417 | 7318 | 1786 | 4195 | 2794 | 6193 | 5345 |
| 3906 | 2254 | 3539 | 2934 | 2904 | 2105 | 7150 | 2219 | 3350 | 4346 |
| 13379 | 1263 | 5829 | 2804 | 1604 | 3235 | 12754 | 3390 | 5918 | 3222 |
| 8103 | 6187 | 1889 | 1437 | 2246 | 1680 | 1491 | 2340 | 3241 | 3371 |
| 10103 | 2643 | 6666 | 3334 | 7787 | 1732 | 6120 | 2504 | 3215 | 6643 |
| 7893 | 7777 | 7890 | 2282 | 6797 | 2389 | 4248 | 2483 | 10147 | 11626 |
| 8872 | 9121 | 5633 | 2719 |  | 4218 | 3925 | 2081 | 1847 | 5389 |
|  |  |  | 2665 |  |  | 9656 | 2240 | 1662 | 7827 |
|  |  |  | 1751 |  |  |  | 8987 |  | 3932 |
|  |  |  | 2048 |  |  |  | 2485 |  | 10547 |
|  |  |  | 6278 |  |  |  | 1445 |  | 3971 |
|  |  |  |  |  |  |  | 2014 |  | 5912 |
|  |  |  |  |  |  |  | 1159 |  | 6095 |
|  |  |  |  |  |  |  | 5833 |  | 6042 |
|  |  |  |  |  |  |  | 1729 |  | 1515 |
|  |  |  |  |  |  |  | 3528 |  | 8767 |
|  |  |  |  |  |  |  | 3837 |  | 6528 |
|  |  |  |  |  |  |  | 8116 |  | 4341 |
|  |  |  |  |  |  |  | 3190 |  | 4522 |
|  |  |  |  |  |  |  |  |  | 1580 |
