# Supplementary material for: Regulation of life span by the gut microbiota in the short-lived African turquoise killifish
Source: eLife. 2017 Aug 22;6:e27014. doi: 10.7554/eLife.27014 (PMC5566455; doi:10.7554/eLife.27014)
Supplement: Figure 5—source data 1. — DOI: http://dx.doi.org/10.7554/eLife.27014.019 [file elife-27014-fig5-data1.docx]

| **Figure 5 – source data 1** | |  |  |  |  |  |
| --- | --- | --- | --- | --- | --- | --- |
| **Beta diversity significance at one week post-transfer** | | | |  |  |  |
|  |  |  |  |  |  |  |
| **Unweighted UniFrac** |  |  |  |  |  |  |
|  | **6wk** | **10wk** | **16wk** | **Ymt** | **Abx** | **Omt** |
| **6wk** | - | 0.014 | 0.001 | 0.004 | 0.001 | 0.002 |
| **10wk** | 0.014 | - | 0.002 | 0.016 | 0.045 | 0.059 |
| **16wk** | 0.001 | 0.002 | - | 0.003 | 0.05 | 0.242 |
| **Ymt** | 0.004 | 0.016 | 0.003 | - | 0.017 | 0.032 |
| **Abx** | 0.001 | 0.045 | 0.05 | 0.017 | - | 0.584 |
| **Omt** | 0.002 | 0.059 | 0.242 | 0.032 | 0.584 | - |
|  |  |  |  |  |  |  |
| **Bray-Curtis** |  |  |  |  |  |  |
|  | **6wk** | **10wk** | **16wk** | **Ymt** | **Abx** | **Omt** |
| **6wk** | - | 0.013 | 0.001 | 0.009 | 0.009 | 0.001 |
| **10wk** | 0.013 | - | 0.142 | 0.009 | 0.465 | 0.792 |
| **16wk** | 0.001 | 0.142 | - | 0.003 | 0.046 | 0.254 |
| **Ymt** | 0.009 | 0.009 | 0.003 | - | 0.007 | 0.007 |
| **Abx** | 0.009 | 0.465 | 0.046 | 0.007 | - | 0.387 |
| **Omt** | 0.001 | 0.792 | 0.254 | 0.007 | 0.387 | - |
