# Supplementary material for: Regulation of life span by the gut microbiota in the short-lived African turquoise killifish
Source: eLife. 2017 Aug 22;6:e27014. doi: 10.7554/eLife.27014 (PMC5566455; doi:10.7554/eLife.27014)
Supplement: Figure 5—source data 2. — DOI: http://dx.doi.org/10.7554/eLife.27014.020 [file elife-27014-fig5-data2.docx]

| **Figure 5 – source data 2** | |  |  | |
| --- | --- | --- | --- | --- |
| **Regression between host lifespan and bacterial genus abundance** | | | |  |
| **Genus** | **P value** | **R-square** | **Adjusted R-square** | |
| *Enterococcus* | 0.003706776 | 0.992600188 | 0.988900282 | |
| *Plesiomonas* | 0.004338397 | 0.991342028 | 0.987013041 | |
| *Aliivibrio* | 0.010432989 | 0.979242869 | 0.968864304 | |
| *Leucobacter* | 0.012433899 | 0.975286804 | 0.962930206 | |
| *Myroides* | 0.015826932 | 0.968596627 | 0.952894941 | |
| *Jeotgalicoccus* | 0.018012821 | 0.96429882 | 0.94644823 | |
| *Marinilactibacillus* | 0.022672523 | 0.955168996 | 0.932753495 | |
| *Exiguobacterium* | 0.023563541 | 0.953428159 | 0.930142239 | |
| *Leuconostoc* | 0.025705124 | 0.949250506 | 0.923875758 | |
| *Psychrobacter* | 0.031568982 | 0.937858637 | 0.906787955 | |
| *Paracoccus* | 0.035230192 | 0.930780783 | 0.896171174 | |
| *Pseudoalteromonas* | 0.036045637 | 0.929208013 | 0.89381202 | |
| *Arcobacter* | 0.043303847 | 0.915267529 | 0.872901293 | |
| *Arthrobacter* | 0.04988702 | 0.902714674 | 0.854072011 | |
| *Microbacterium* | 0.051662884 | 0.899343285 | 0.849014928 | |
| *Chryseobacterium* | 0.057383533 | 0.888525803 | 0.832788705 | |
| *Halomonas* | 0.060114797 | 0.883384194 | 0.825076291 | |
| *Rhodobacter* | 0.06169409 | 0.880417981 | 0.820626972 | |
| *Planococcus* | 0.062524949 | 0.878859472 | 0.818289208 | |
| *Propionigenium* | 0.071110977 | 0.862834817 | 0.794252226 | |
| *Brachybacterium* | 0.073041022 | 0.859252947 | 0.788879421 | |
| *Dietzia* | 0.073041022 | 0.859252947 | 0.788879421 | |
| *Microbispora* | 0.073041022 | 0.859252947 | 0.788879421 | |
| *Fluviicola* | 0.073041022 | 0.859252947 | 0.788879421 | |
| *Sphingobacterium* | 0.073041022 | 0.859252947 | 0.788879421 | |
| *Brochothrix* | 0.073041022 | 0.859252947 | 0.788879421 | |
| *Planomicrobium* | 0.073041022 | 0.859252947 | 0.788879421 | |
| *Salinicoccus* | 0.073041022 | 0.859252947 | 0.788879421 | |
| *Psychrilyobacter* | 0.073041022 | 0.859252947 | 0.788879421 | |
| *Nitratireductor* | 0.073041022 | 0.859252947 | 0.788879421 | |
| *Ruegeria* | 0.073041022 | 0.859252947 | 0.788879421 | |
| *Chitinilyticum* | 0.073041022 | 0.859252947 | 0.788879421 | |
| *Pseudidiomarina* | 0.073041022 | 0.859252947 | 0.788879421 | |
| *Serratia* | 0.073041022 | 0.859252947 | 0.788879421 | |
| *Proteiniclasticum* | 0.088315186 | 0.831169199 | 0.746753799 | |
| *Carnobacterium* | 0.098165569 | 0.813305341 | 0.719958011 | |
| *Flavobacterium* | 0.155189663 | 0.713704505 | 0.570556758 | |
| *Acinetobacter* | 0.207527739 | 0.628012284 | 0.442018426 | |
| *Citrobacter* | 0.211183494 | 0.62223148 | 0.433347221 | |
| *Agrobacterium* | 0.236396917 | 0.583089668 | 0.374634502 | |
| *Morganella* | 0.301990408 | 0.48721739 | 0.230826085 | |
| *Lactococcus* | 0.349691759 | 0.422900808 | 0.134351212 | |
| *Delftia* | 0.366005293 | 0.401949288 | 0.102923933 | |
| *Vibrio* | 0.382646818 | 0.381124952 | 0.071687427 | |
| *Mycobacterium* | 0.399926465 | 0.360088247 | 0.040132371 | |
| *Pseudomonas* | 0.403033181 | 0.356369383 | 0.034554075 | |
| *Propionibacterium* | 0.421686013 | 0.334447068 | 0.001670602 | |
| *Leptothrix* | 0.427214853 | 0.328082824 | -0.007875764 | |
| *Salinivibrio* | 0.44411694 | 0.309005976 | -0.036491036 | |
| *Enhydrobacter* | 0.445172896 | 0.307833115 | -0.038250327 | |
| *Sphingomonas* | 0.473719587 | 0.276971074 | -0.08454339 | |
| *Photobacterium* | 0.517885801 | 0.232434101 | -0.151348849 | |
| *Vogesella* | 0.585140856 | 0.17210811 | -0.241837836 | |
| *Proteus* | 0.586163383 | 0.171260746 | -0.243108881 | |
| *Micrococcus* | 0.587043396 | 0.170533157 | -0.244200265 | |
| *Marinobacter* | 0.605497745 | 0.155632029 | -0.266551957 | |
| *Stenotrophomonas* | 0.610275233 | 0.151885394 | -0.272171909 | |
| *Granulicatella* | 0.623679614 | 0.141617033 | -0.28757445 | |
| *Bacillus* | 0.637296719 | 0.13155367 | -0.302669495 | |
| *Ochrobactrum* | 0.678456568 | 0.103390179 | -0.344914732 | |
| *Streptococcus* | 0.699081754 | 0.090551791 | -0.364172313 | |
| *Shewanella* | 0.704707995 | 0.087197368 | -0.369203948 | |
| *Klebsiella* | 0.715529319 | 0.080923568 | -0.378614648 | |
| *Staphylococcus* | 0.734480665 | 0.070500517 | -0.394249224 | |
| *Corynebacterium* | 0.761593239 | 0.056837784 | -0.414743324 | |
| *Vagococcus* | 0.922407082 | 0.006020661 | -0.490969009 | |
| *Aequorivita* | 0.926772679 | 0.00536224 | -0.491956639 | |
| *Brumimicrobium* | 0.937474235 | 0.003909471 | -0.494135793 | |
| *Candidatus Portiera* | 0.964841828 | 0.001236097 | -0.498145854 | |
| *Arenibacter* | 0.966292401 | 0.001136202 | -0.498295697 | |
| *Gelidibacter* | 0.966292401 | 0.001136202 | -0.498295697 | |
| *Winogradskyella* | 0.966292401 | 0.001136202 | -0.498295697 | |
| *Natronobacillus* | 0.966292401 | 0.001136202 | -0.498295697 | |
| *Clostridiisalibacter* | 0.966292401 | 0.001136202 | -0.498295697 | |
| *Peptoniphilus* | 0.966292401 | 0.001136202 | -0.498295697 | |
| *Limnobacter* | 0.966292401 | 0.001136202 | -0.498295697 | |
| *Neisseria* | 0.966292401 | 0.001136202 | -0.498295697 | |
| *Oceanimonas* | 0.966292401 | 0.001136202 | -0.498295697 | |
| *Alkalimonas* | 0.966292401 | 0.001136202 | -0.498295697 | |
| *Rheinheimera* | 0.966292401 | 0.001136202 | -0.498295697 | |
| *Providencia* | 0.998183343 | 3.30E-06 | -0.49999505 | |
