# Supplementary material for: Regulation of life span by the gut microbiota in the short-lived African turquoise killifish
Source: eLife. 2017 Aug 22;6:e27014. doi: 10.7554/eLife.27014 (PMC5566455; doi:10.7554/eLife.27014)
Supplement: Figure 6—source data 1. — DOI: http://dx.doi.org/10.7554/eLife.27014.023 [file elife-27014-fig6-data1.docx]

| **Figure 6 – source data 1** | |  |  |  |  |
| --- | --- | --- | --- | --- | --- |
| **Network hubs of OTU-based networks** | | |  |  |  |
| **Bacterial genus name** | **6wk** | **16wk** | **Ymt** | **Abx** | **Omt** |
| **Corynebacterium** | YES | NO | NO | NO | NO |
| **Dietzia** | YES | NO | NO | NO | NO |
| **Microbacterium** | YES | NO | NO | NO | NO |
| **Exiguobacterium** | YES | NO | YES | NO | NO |
| **Enterococcus** | YES | NO | NO | NO | NO |
| **Paracoccus** | YES | NO | NO | NO | NO |
| **Rhodobacter** | YES | NO | NO | NO | NO |
| **Ruegeria** | YES | NO | NO | NO | NO |
| **Photobacterium** | YES | NO | NO | NO | NO |
| **Arthrobacter** | YES | NO | NO | NO | NO |
| **Chryseobacterium** | YES | NO | NO | NO | NO |
| **Planococcus** | YES | NO | YES | NO | NO |
| **Carnobacterium** | YES | NO | NO | NO | NO |
| **Propionigenium** | YES | NO | YES | NO | NO |
| **Halomonas** | YES | NO | NO | NO | NO |
| **Psychrobacter** | YES | NO | YES | NO | NO |
| **Propionibacterium** | NO | YES | YES | YES | NO |
| **Delftia** | NO | YES | YES | YES | NO |
| **Vibrio** | NO | YES | NO | NO | YES |
| **Lactococcus** | NO | YES | NO | YES | NO |
| **Vogesella** | NO | YES | NO | NO | YES |
| **Citrobacter** | NO | YES | YES | YES | NO |
| **Morganella** | NO | YES | NO | NO | NO |
| **Acinetobacter** | NO | YES | NO | YES | NO |
