# Supplementary material for: Regulation of life span by the gut microbiota in the short-lived African turquoise killifish
Source: eLife. 2017 Aug 22;6:e27014. doi: 10.7554/eLife.27014 (PMC5566455; doi:10.7554/eLife.27014)
Supplement: Figure 7—source data 1. — DOI: http://dx.doi.org/10.7554/eLife.27014.025 [file elife-27014-fig7-data1.docx]

| **Figure 7 – source data 1** | |  |  |
| --- | --- | --- | --- |
| **Best hits from zebrafish pBlast on DEGs not annotated in the Valenzano et al. 2015 genome paper** | | | |
| **Comparison** | **Group** | **Gene name** | **pBlast D.rerio best hit** |
| 16wk versus 6wk | 6wk | MFAP4-mRNA-1 | MFAP4 |
|  | 6wk | MFAP4-mRNA-2 | MFAP4 |
|  | 6wk | C14ORF39-mRNA-1 | - |
|  | 16wk | NFURG05812010005-mRNA-1 | HAMP |
| 16wk versus Ymt | Ymt | MFAP4-mRNA-1 | MFAP4 |
|  | Ymt | MFAP4-mRNA-2 | MFAP4 |
|  | 16wk | NFURLNR02806021000-RNA-1 | - |
|  | 16wk | NFURLNR02791010010-RNA-1 | - |
| Omt versus Ymt | Ymt | POU2AF1-mRNA-1 | POU2AF1 |
|  | Ymt | IL4I1(2of3)-mRNA-1 | IL4I1 |
|  | Ymt | NFURG05812010005 | HAMP |
|  | Omt | TBT-BP1(1of2)-mRNA-1 | - |
|  | Omt | NFURG14715010300-mRNA-1 | - |
| Omt + 16wk versus Ymt | Ymt | NFURG05812010005-mRNA-1 | HAMP |
|  | Ymt | IL4I1(2of3)-mRNA-1 | IL4I1 |
|  | Ymt | IL5RA-mRNA-1 | IL2RGA |
|  | Ymt | POU2AF1-mRNA-1 | POU2AF1 |
|  | Omt+16wk | DAZ2-mRNA-1 | - |
| Omt+16wk vs. 6wk+Ymt | 6wk+Ymt | MFAP4-mRNA-1 | MFAP4 |
|  | 6wk+Ymt | MFAP4-mRNA-2 | MFAP4 |
|  | Omt+16wk | DAZ2-mRNA-1 | - |
| Omt vs 6wk | Omt | TSPAN8(1of4) | TSPAN25,CD53 |
|  | Omt | NFURG05812010005-mRNA-1 | HAMP |
|  | 6wk | MFAP4-mRNA-1 | MFAP4 |
|  | 6wk | MFAP4-mRNA-2 | MFAP4 |
| Ymt vs 6wk | Ymt | NFURG05812010005-mRNA-1 | HAMP |
|  | Ymt | F11-mRNA-1 | - |
|  | 6wk | S100PBP-mRNA-1 | KOP / ASKOPOS |
